# Practicing procedural skills in clinical settings: A medical student’s perspective

**DOI:** 10.30476/jamp.2020.86425.1237

**Published:** 2021-04

**Authors:** ZOYA MEHDI, LAILA HAZARA, KHULAT SAQI, SAMINEH YOUSEFI

**Affiliations:** 1 Medical School, Faculty of Life Sciences and Medicine, King's College London, London, UK

**Dear Editor**

We read with great interest ‘Exploring undergraduate medical students’ perception of learning procedural skills and its outcomes in clinical settings’ ( 1 ) as third year medical students at King’s college London. We would like to share our experience with practicing procedural skills and make suggestions to help bridge the gap between learning and skill acquisition in a hospital. 

Graduates are expected to show competence in 32 core practical skills and procedures in accordance with the GMC guidelines ( 2 ). This requires doctors to demonstrate confidence to perform the task safely and effectively. However, we believe that there is a lack of opportunity to practice skills on patients in hospital preventing students from maximising their potential and competency.

King’s College London gives students early exposure to the hospital setting from the second year of the course. This enables students to develop confidence and familiarity within the clinical environment. Transitioning into clinical placements earlier has shown to have a better outcome on overall performance ( 3 ). Additionally, this allows students to learn directly from experience to deepen their understanding of different procedures and skills. We believe that this should be adopted by all medical schools as it encourages more patient contact and more opportunities to practice the required skills. 

Furthermore, students are given portfolios each year to monitor their clinical progress at hospital. This includes a section of core clinical skills which are required to be performed sufficiently. The use of portfolios is associated with improved performance in comparison to using logbooks ( 4 ). The portfolio can be signed off by any clinician who is competent to perform the skill and has supervised us to complete the task accurately. It includes a description of the steps and the skill level required from us at that stage. This enables us and clinicians at hospital to gain a better understanding of what is expected from students. All the tasks in the clinical skill record must be completed satisfactorily to progress to the next year of the course. Personally, this encourages us to self-direct our learning by actively seeking out assistance to translate our knowledge into practice. Although this can be a challenge, it also ensures that we graduate with appropriate practice of clinical and procedural skills.

Additionally, students are concerned about compromising patient care for the purpose of learning, and this can prevent them from practicing procedural skills. To overcome this, our hospital sites offer students to work closely alongside the team to arrange book-in sessions to practice procedures under supervision in clinical skill rooms with the use of models. This allows us to gain feedback and improve our technique ultimately increasing our confidence in our ability. As the session is based at the hospital, we can turn to practice, immediately after the session, on a patient to consolidate the skill. This is effective as higher levels of confidence have a positive correlation with performance, thus improving patient experience ( 5 ). 

Overall, practicing procedures in clinical settings is fundamental to developing students’ abilities and cannot be taught through skill labs alone. We recommend that these approaches highlighted above alongside the suggestions in this paper ( 1 ) be incorporated into medical training to further help medical students develop into competent and confident doctors. 
